# Supplementary material for: Oxygen vacancy associated single-electron transfer for photofixation of CO2 to long-chain chemicals
Source: Nat Commun. 2019 Feb 15;10:788. doi: 10.1038/s41467-019-08697-x (PMC6377667; doi:10.1038/s41467-019-08697-x)
Supplement: Supplementary file 1 — Supplementary Information [file 41467_2019_8697_MOESM1_ESM.pdf]

Oxygen vacancy associated single-electron transfer for photofixation of CO_2 to long-chain chemicals

Chen et al.

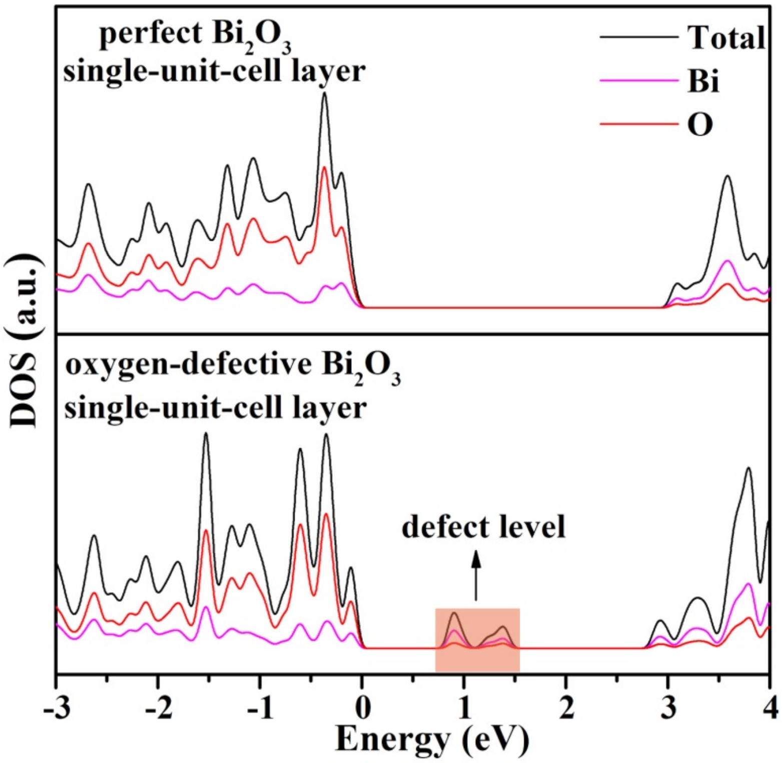

Supplementary Figure 1. Calculated density of states of perfect and oxygen-defective Bi_2O_3 single-unit-cell slab.

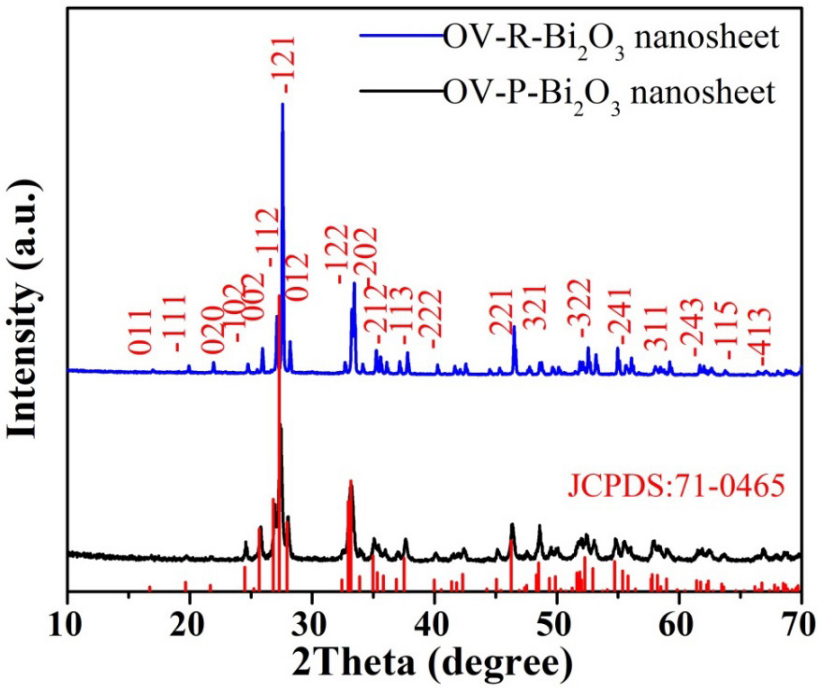

Supplementary Figure 2. XRD patterns of the Bi_2O_3 nanosheets with rich and poor oxygen vacancies.

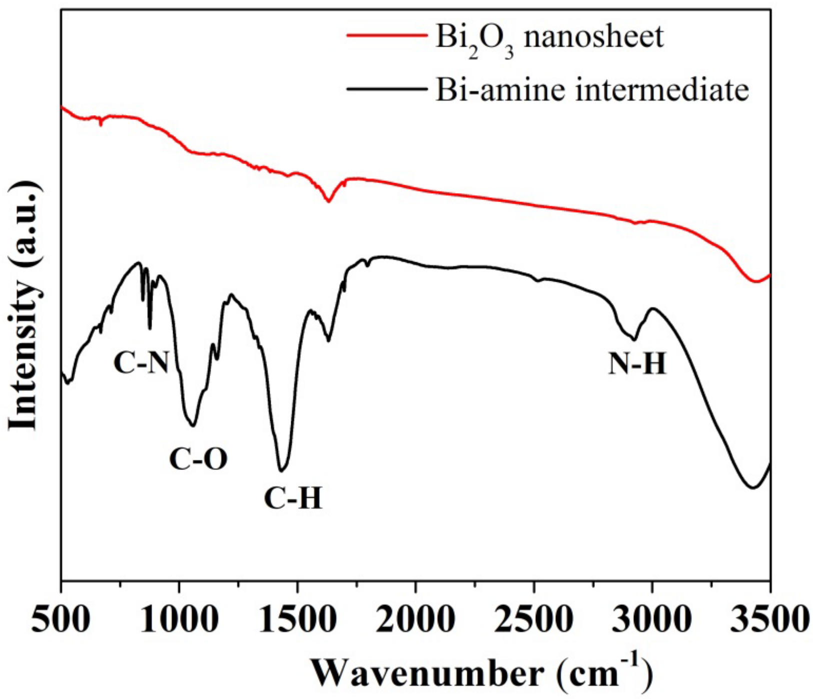

Supplementary Figure 3. IR spectra of Bi-amine hybrid intermediate and Bi_2O_3 nanosheets. The vibration bands suggest the existence of amine in Bi-amine hybrid intermediate. After ultrasonication and exfoliation, the vibration bands of amine are absent, indicating the clean surface of the as-prepared Bi_2O_3 nanosheets

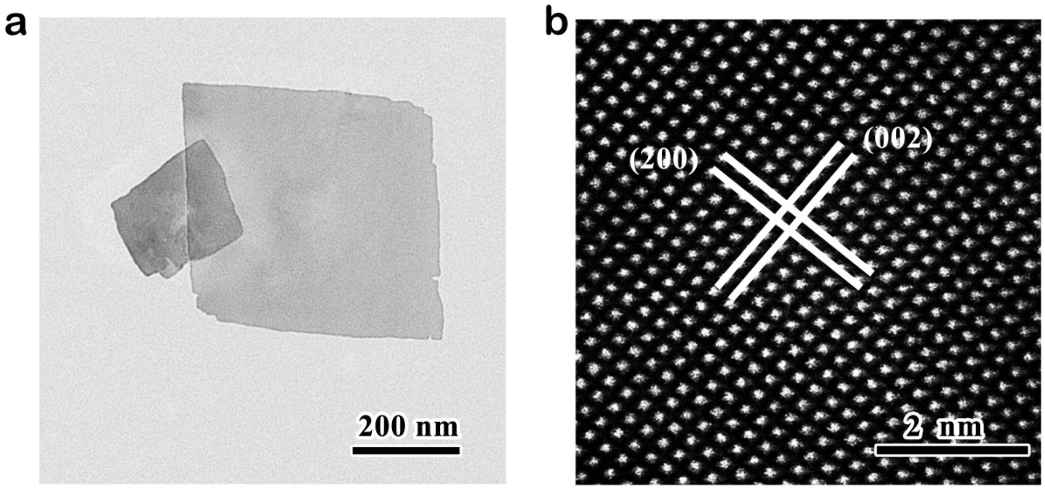

Supplementary Figure 4. (a) TEM image, and (b) atomic-resolution HAADF-STEM image of OV-P-Bi_2O_3 nanosheets.

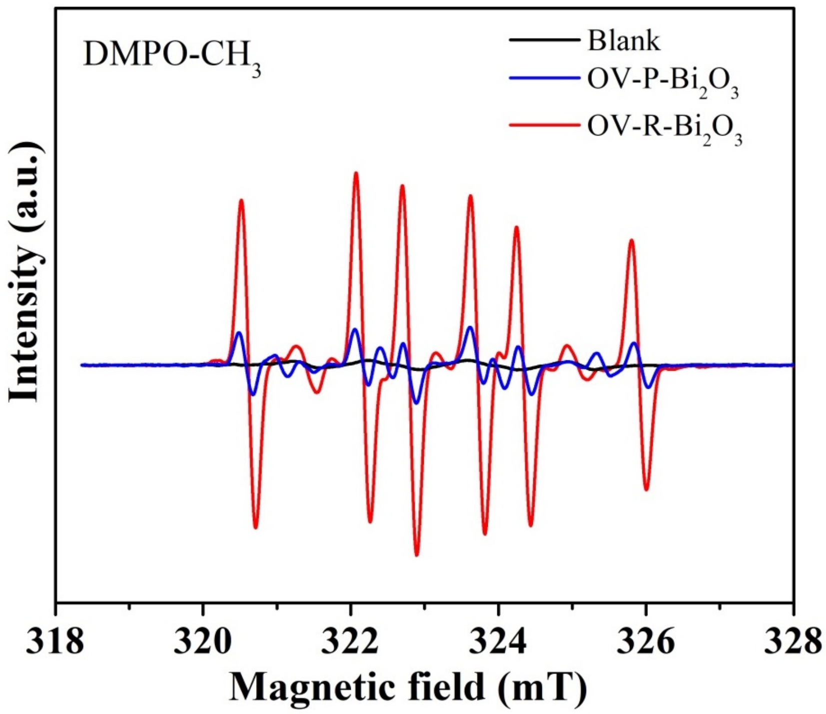

Supplementary Figure 5. *In-situ* ESR of DMPO-CH_3, where DMPO was used as the spin trapping agent.

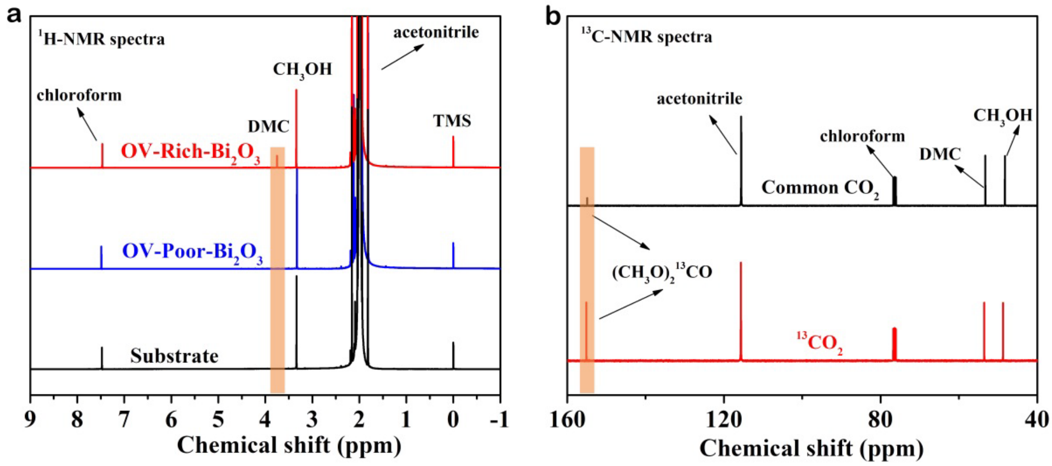

Supplementary Figure 6. Representative NMR spectra of synthetic DMC from the reaction of CO_2 and CH_3OH in acetonitrile (CH_3CN) solution. (a) ^{1}H-NMR spectra of the products after 8 h CO_2 reaction. (b) ^{13}C-NMR spectra of the products using common CO_2 and ^{13}C isotopic labeling CO_2.

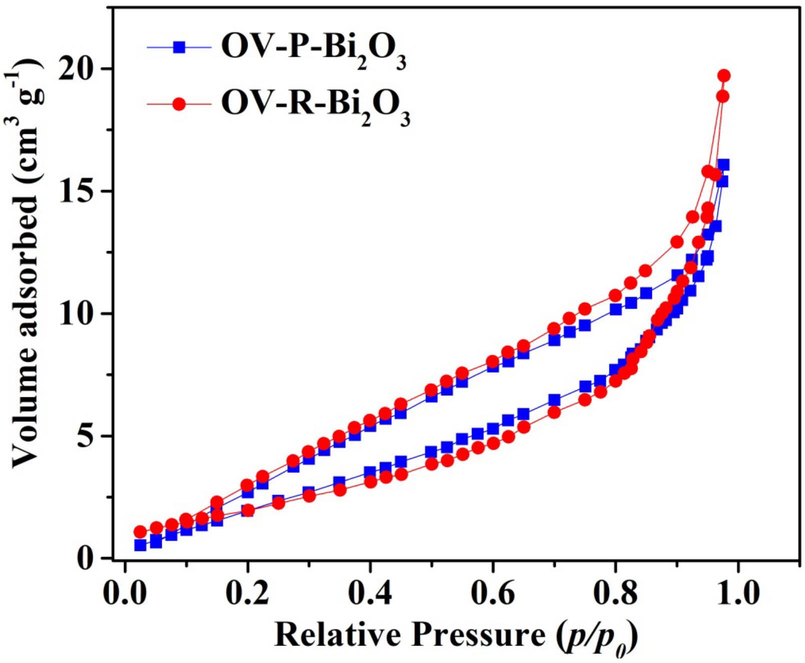

Supplementary Figure 7. BET surface area of OV-R-Bi_2O_3 and OV-P-Bi_2O_3 samples. The BET surface areas of OV-R-Bi_2O_3 and OV-P-Bi_2O_3 were 30.18 m^2 g^{-1} and 28.96 m^2 g^{-1}, respectively, which suggests similar BET surface area of above samples.

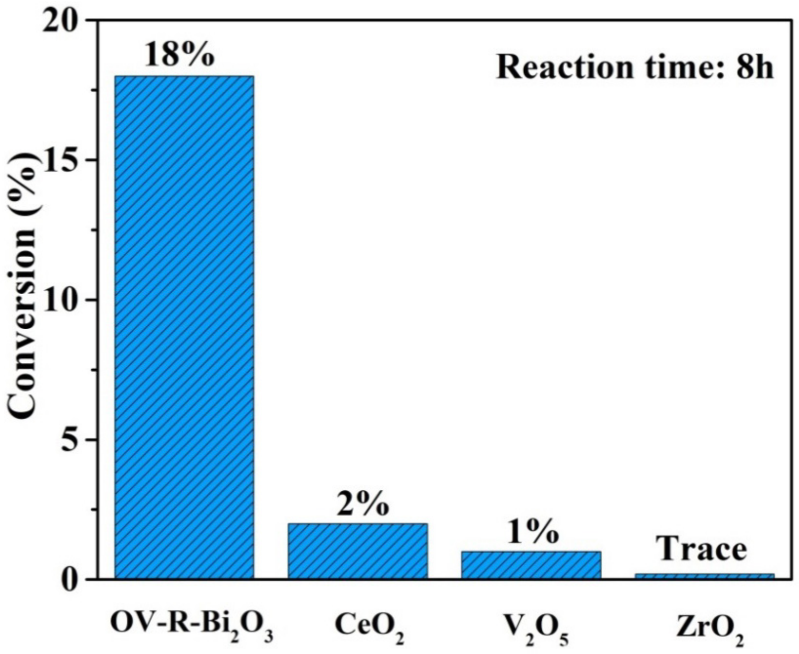

Supplementary Figure 8. Performances of various catalysts for CO_2 fixation to DMC under the same reaction conditions (CO_2 (0.2 Mpa) at 373 K under Xe-lamp irradiation). Herein, the CeO_2 nanosheets, V_2O_5 nanosheets, and ZrO_2 nanoparticles are prepared according to literatures[1-3].

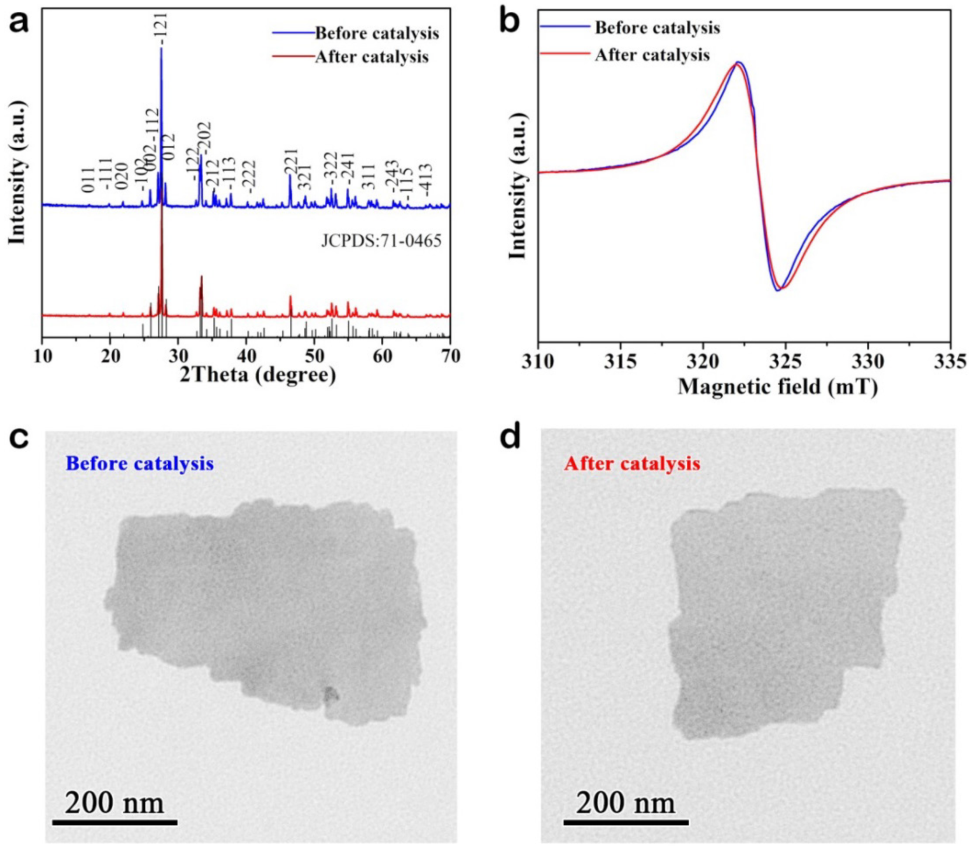

Supplementary Figure 9. Characterization of the OV-R-Bi_2O_3 sample after 25 catalytic cycles. (a) XRD pattern, (b) Room temperature ESR spectra, (c, d) TEM image before and after catalytic cycles up to 200 hours. The above results show that the crystal phase, morphology and vacancy concentration of OV-R-Bi_2O_3 were well maintained after 25 catalytic cycles.

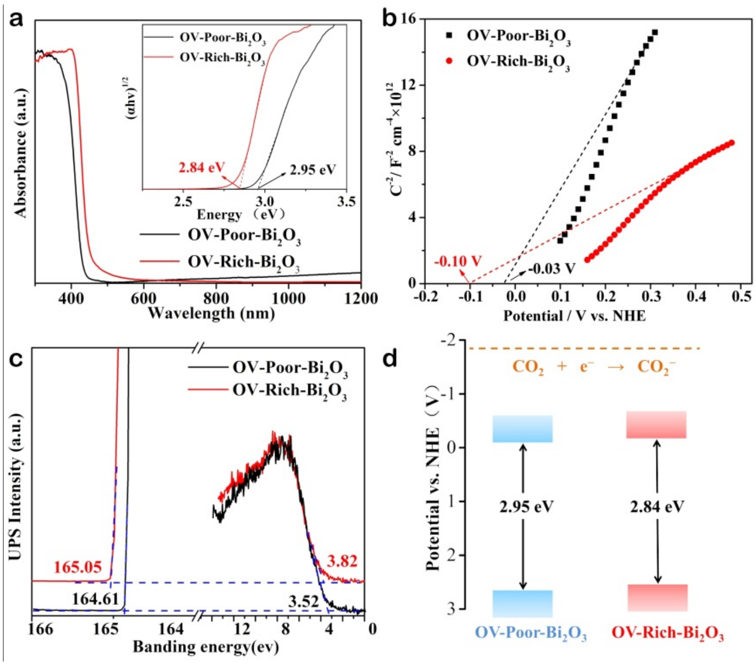

Supplementary Figure 10. Band structure study. (a) UV-vis spectra. Inset: corresponding Tauc plots of samples. (b) Mott-Schottky curves. (c) Valence-band spectra and Secondary electron cutoff ($E_{cut-off}$) measured by SRPES. (d) Band structure diagram. Based on the Mott-Schottky curves, the flat band potentials, which are close to the corresponding conduction band minima (CBMs) in n-type semiconductors, were determined to be −0.10 and −0.03 eV (vs. normal hydrogen electrode (NHE)) for OV-Rich-Bi_2O_3 and OV-Poor-Bi_2O_3 nanosheets, respectively. In addition, the valence band maxima (VBMs) of Bi_2O_3 nanosheets were resolved by synchrotron radiation photoelectron spectroscopy (SRPES) with excitation light (hv = 168.4 eV)[4,5]. As demonstrated, the VBM of OV-Rich-Bi_2O_3 and OV-Poor-Bi_2O_3 nanosheets were estimated to be about −7.17 and −7.31 eV vs evac (vacuum level). With the combination of SRPES and UV–vis results, the CBMs of OV-Rich-Bi_2O_3 and OV-Poor-Bi_2O_3 nanosheets were estimated to be −0.11 and −0.08 eV (vs. NHE), in good agreement with the values estimated from Mott-Schottky curves. It is worth noting that the SRPES spectroscopy is more suitable for determining the band structures, as compared to the electrochemical method[6].

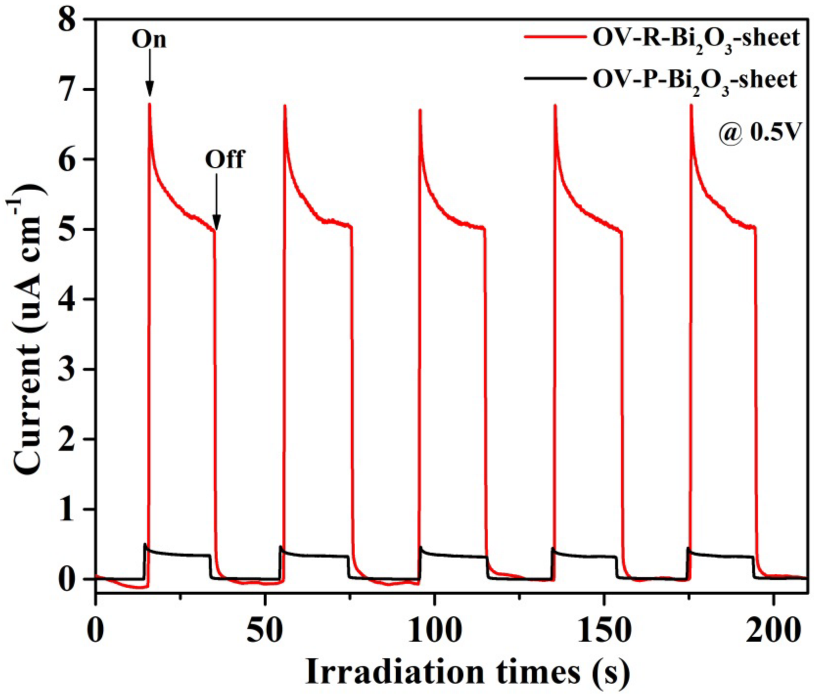

Supplementary Figure 11. Photocurrent response of Bi_2O_3 nanosheets with rich and poor oxygen vacancies, respectively. The photocurrent-voltage curves suggest enhanced photocurrent response under negative bias, confirming the enhanced photogeneration of hot electrons in the ultrathin Bi_2O_3-sheets with rich defects.

Supplementary Table 1. Direct synthesis of DMC from CO_2 and CH_3OH with different catalysts.

Catalyst	Conversion yield[a] (%)	Selectivity[b] (%)	Reaction condition	Ref.
**Bi_2O_3-bulk**	Trace	Trace	0.2 Mpa, 373K,light, 8h	This work
**OV-P-Bi_2O_3 nanosheet**	2	>99	0.2 Mpa, 373K,light, 8h	This work
**OV-R-Bi_2O_3 nanosheet**	**18**	**>99**	0.2 Mpa, 373K,light, 8h	This work
ZrO_2-KCl-K_2CO_3	10.8	43.2	9.5 Mpa, 423K, 8h	7
$Mg(OCH_3)_2$ + ZrO_2-KCl	3.9	45.6	9.5 Mpa, 423K, 8h	7
H_3PO_4/V_2O_5	1.96	93.1	0.6 Mpa, 413K, 8h	8
$H_3PW_{12}O_{40}/ZrO_2$	2.02	>99	7.6 Mpa, 373K, 7h	9
CeO_2- ZrO_2	0.8	>99	6 Mpa, 383K, 24h	10
$Co_{1.5}PW_{12}O_{40}$	3.8	65.2	0.5 Mpa, 373K, 5h	11
Mo-Cu-Fe/SiO_2	7	88	0.5 Mpa, 393K, 4h	12

[a]Determined by NMR analyses, using s-trioxane as the internal standard, mol %

[b]selectivity = $yield_{DMC}$*2/$conversion_{methanol}$

Supplementary References

1 Sun, Y., Liu, Q., Gao, S., Cheng, H., Lei, F., Sun, Z., Jiang, Y., Su, H., Wei, S. & Xie, Y. Pits confined in ultrathin cerium(IV) oxide for studying catalytic centers in carbon monoxide oxidation. *Nature Commun.* **4**, 2899 (2013).

2 Peng, X., Zhang, X., Wang, L., Hu, L., Cheng, S. H.-S., Huang, C., Gao, B., Ma, F., Huo, K. & Chu, P. K. Hydrogenated V_2O_5Nanosheets for Superior Lithium Storage Properties. *Adv. Funct. Mater.* **26**, 784-791 (2016).

3 Fakhri, A., Behrouz, S., Tyagi, I., Agarwal, S. & Gupta, V. K. Synthesis and characterization of ZrO_2 and carbon-doped ZrO_2 nanoparticles for photocatalytic application. *J. Mol. Liq.* **216**, 342-346 (2016).

4 Zhang, N., Li, X., Ye, H., Chen, S., Ju, H., Liu, D., Lin, Y., Ye, W., Wang, C., Xu, Q., Zhu, J., Song, L., Jiang, J. & Xiong, Y. Oxide Defect Engineering Enables to Couple Solar Energy into Oxygen Activation. *J. Am. Chem. Soc.* **138**, 8928-8935 (2016).

5 Liu, W., Cao, L., Cheng, W., Cao, Y., Liu, X., Zhang, W., Mou, X., Jin, L., Zheng, X., Che, W., Liu, Q., Yao, T. & Wei, S. Single-Site Active Cobalt-Based Photocatalyst with a Long Carrier Lifetime for Spontaneous Overall Water Splitting. *Angew. Chem. Int. Ed.* **56**, 9312-9317 (2017).

6 Liu, J., Liu, Y., Liu, N., Han, Y., Zhang, X., Huang, H., Lifshitz, Y., Lee, S. T., Zhong, J. & Kang, Z. Water splitting. Metal-free efficient photocatalyst for stable visible water splitting via a two-electron pathway. *Science* **347**, 970-974 (2015).

7 Eta, V., Mäki-Arvela, P. i., Leino, A.-R., Kordás, K. n., Salmi, T., Murzin, D. Y. & Mikkola, J.-P. Synthesis of Dimethyl Carbonate from Methanol and Carbon Dioxide: Circumventing Thermodynamic Limitations. *Ind. Edg. Chem. Res.* **49**, 9609-9617 (2010).

8 Wu, X. L., Xiao, M., Meng, Y. Z. & Lu, Y. X. Direct synthesis of dimethyl carbonate on H_3PO_4 modified V_2O_5. *J. Mol. Catal. A: Chem.* **238**, 158-162 (2005).

9 Jiang, C., Guo, Y., Wang, C., Hu, C., Wu, Y. & Wang, E. Synthesis of dimethyl carbonate from methanol and carbon dioxide in the presence of polyoxometalates under mild conditions. *Appl. Catal., A* **256**, 203-212 (2003).

10 Tomishige, K. & Kunimori, K. Catalytic and direct synthesis of dimethyl

carbonate starting from carbon dioxide using CeO_2-ZrO_2 solid solution heterogeneous catalyst: effect of H2O removal from the reaction system. *Appl Catal a-Gen* **237**, 103-109 (2002).

11 Aouissi, A., Apblett, A. W., Al-Othman, Z. A. & Al-Amro, A. Direct synthesis of dimethyl carbonate from methanol and carbon dioxide using heteropolyoxometalates: the effects of cation and addenda atoms. *Transition Met. Chem.* **35**, 927-931 (2010).

12 Zhou, Y.-J., Xiao, M., Wang, S.-J., Han, D.-M., Lu, Y.-X. & Meng, Y.-Z. Effects of Mo promoters on the Cu–Fe bimetal catalysts for the DMC formation from CO_2 and methanol. *Chin. Chem. Lett.* **24**, 307-310 (2013).
